# Cavitation noise suppression in automotive heat pumps via vapor-doped supercavitation

**DOI:** 10.1371/journal.pone.0325719

**Published:** 2025-07-02

**Authors:** Li Hu, Yachao Qin, Bing Ruan, Yeqing Wan, Bo Wang, Ruili Tian, Ni Quan

**Affiliations:** 1 School of Automobile and Traffic Engineering, Wuhan University of Science and Technology, Wuhan, China; 2 Manager Department, Automotive Engineering Corporation, Tianjin, China; 3 Technical Department, Scivic Engineering Corporation, Luoyang, China; 4 Dongfeng Mahle Thermal System Co., Ltd., Wuhan, China; Monash University, AUSTRALIA

## Abstract

Aiming at two-phase flow-induced noise of refrigerant in automotive heat pump air conditioner, the influence of the changes of pressure, flow rate and gas-phase volume fraction on the acoustic radiation produced by refrigerant in the process of phase change of electronic expansion valve is analyzed by constructing the refrigerant cavitation model and acoustic radiation model. It is found that the sudden pressure drop of the refrigerant leads to the phase change, forming the cavitation phenomenon, which in turn generates noise. Through CFD numerical simulation, the characteristics of the flow field distribution under different working conditions are explored, and the correlation between thermodynamic parameters and acoustic parameters is revealed. In order to reduce the noise, proposing vapor-doped supercavitation optimization strategy to study the influence of the structural parameters of the vapor-doped components on cavitation and noise, and the key structural parameters are optimized by the response surface methodology, and the peak sound pressure level is reduced by 9.95 dB, and the effective sound pressure level RMS is reduced by 0.79 dB. The results show that this component significantly reduce the SPL, which provides a theoretical basis and practical guidance for the noise reduction design of automotive heat pump air conditioner.

## 1 Introduction

The advancement of the automotive industry and the rising environmental standards have led to the widespread adoption of heat pump automotive air conditioners in the domain of new energy vehicles. This is due to the high efficiency and energy-saving capabilities of these devices [1]. As a core component, the performance of electronic expansion valve is related to the efficiency of air conditioning and system stability. However, two-phase flow noise is generated during operation, which both disturbs passengers and threatens the safety of components. Therefore, the study of two-phase flow noise mechanism and noise reduction method of electronic expansion valve is crucial to improve the performance of heat pump automotive air conditioning.

When the electronic expansion valve regulates the refrigerant flow, a phase change occurs within the refrigerant as it passes through the valve, resulting in a two-phase flow comprising both a gas and a liquid. During the flow process, rapid changes in flow rate, pressure, temperature, and other parameters give rise to turbulence, vortices, and other complex flow phenomena, which, in turn, induce noise. Additionally, the valve design, material selection, and manufacturing process have a considerable influence on the SPL.

Rodarte et al.[2] investigated the noise issue associated with thermal expansion valves, focusing on the noise generated by the pipe wall downstream of the expansion valve. The noise generated by the expansion valve, which is transmitted through the pipe wall by vibrations and subsequently amplified by the evaporator, is then transmitted to the vehicle. Recent studies have highlighted the complexity of noise generation in automotive heat pump systems. For instance, Chen et al.[3] demonstrated that bubble dynamics, including nucleation, growth, and collapse in refrigerant flow through expansion valves, are critical contributors to high-frequency noise (6–10 kHz) due to pressure pulsations and turbulence interactions. Similarly, Mondal et al.[4] used CFD with the VOF model to analyze silicone oil–air and water–air flows in vertical pipes, revealing that transitions from bubbly to annular flow regimes significantly alter radial void fraction distributions and acoustic energy spectra, emphasizing the role of fluid properties in noise generation.

To reduce the two-phase flow noise associated with the electronic expansion valve, scholars have conducted extensive research. On the one hand, optimize the valve structure and process to reduce flow resistance, reduce turbulence and reduce noise. On the other hand, the use of acoustic principles, take muffling and sound insulation measures to control noise. Zhu et al.[5] introduced a dual-modality electrical sensor to simultaneously measure solid concentration and moisture in gas-solid flows, offering a framework for real-time monitoring of noise-critical parameters in automotive systems. Duan et al.[6] proposed the addition of a groove structure in the expansion valve piping combined with the valve seat chamfering treatment to reduce the noise in the cooling and heating directions by 2.47 dB, respectively, but did not address the multi-parameter coupling effect under the dynamic operating conditions. An automotive air-conditioning experiment [7] verified three muffling schemes: small-aperture mufflers can eliminate high-frequency noises above 9 kHz, the valve-ball-opening scheme makes the noise above 8 kHz basically eliminated, and the expansion muffler improves the subjective perception through the consumption of acoustic energy, but the traditional muffler may sacrifice the efficiency of the system.

The study of vapor-doped corrosion reduction is a more prevalent area of research within the field of hydrodynamics. For instance, Peterka [8] found that 0.4% to 0.7% of the gas can be doped to reduce cavitation cavitation noise in the pipeline, doped 7% of the gas when the noise almost disappeared. Many researchers have suggested that the wall near 1% to 2% of the air concentration can reduce cavitation, 5% to 7% can eliminate cavitation [9]. The concentration of insufflated gas to inhibit cavitation depends on the specimen material. Rasmussen [10] suggested an aeration rate of 1% to avoid the cavitation erosion of an aluminum alloy specimen, while Russell and Sheehan [11] reported that an air concentration of 5.7%was suitable for a concrete specimen. The pressure in the cavitation region of a high-velocity flow with aeration increases because of the formation of a compression wave after the flow has been aerated [12]. Hyung Suk Han et al.[13] studied the refrigerant flow pattern and bubble characteristics at the inlet of the evaporator and discussed the relationship between refrigerant noise and bubble characteristics. Yubo Xia [14] et al. established a transition tube with different structures between the capillary and the evaporator and experimentally studied the influence of refrigerant flow state in the transition tube on the refrigerator noise. The aeration mode can be determined as either self-aeration or forced aeration. Self-aeration uses negative pressure to achieve natural aeration [15], such as in spillway aeration [16] and lock water valve aeration [17]. Huang [18] further pointed out that vapor-doped can increase the local pressure, and even a small amount of vapor-doped can significantly reduce the pressure at the time of vacuole collapse. Wang [19] et al. employed the theory of vacuole dynamics to develop vapor-doped overtubes in capillary tubes and evaporators. The utilization of vapor-doped structures markedly diminished the occurrence of bubble burst noise, and the refrigeration throttling noise was reduced by 1.5 dB. It is therefore reasonable and feasible to introduce the vapor-doped supercavitation method to suppress the cavitation noise. This method involves introducing refrigerant vapor through a vapor-doping structure, thereby increasing the gas content and creating a supercavitation flow [20].

Despite the existence of studies on the suppression of cavitation noise through vapor-doped, the majority of existing methods concentrate on general fluid machinery or static conditions. There remains a paucity of systematic research on the noise mechanism and optimization strategy of electronic expansion valves for automotive heat pumps and air conditioners under dynamic conditions. Furthermore, traditional sound insulation or single-parameter optimization has limited efficacy in complex gas-liquid two-phase flow scenarios, and it is difficult to balance noise suppression and system efficiency. Additionally, there is a paucity of active regulation solutions for gas-liquid two-phase flow, and the optimal design of multi-parameter coupling has not been fully explored.

In this study, we propose a novel approach that combines the vapor-doped supercavitation technique with the response surface method of multi-objective optimization. This approach is applied to the noise control of electronic expansion valves. The regulation of gas-liquid two-phase flow, the suppression of cavitation bubble rupture, and the integration of numerical simulation and experimental verification are pivotal in achieving the precise optimization of key structural parameters. In comparison with extant studies, this method not only solves the noise problem of automotive air conditioning under dynamic working conditions, but also provides a new idea for multidisciplinary cross-optimization (fluid dynamics, acoustics and design methodology), which is of significant value for engineering applications.

## 2 Study on the mechanism of flow-induced noise in the electronic expansion valve

### 2.1 Mechanism of flow-induced noise generation

**Fig 1:**
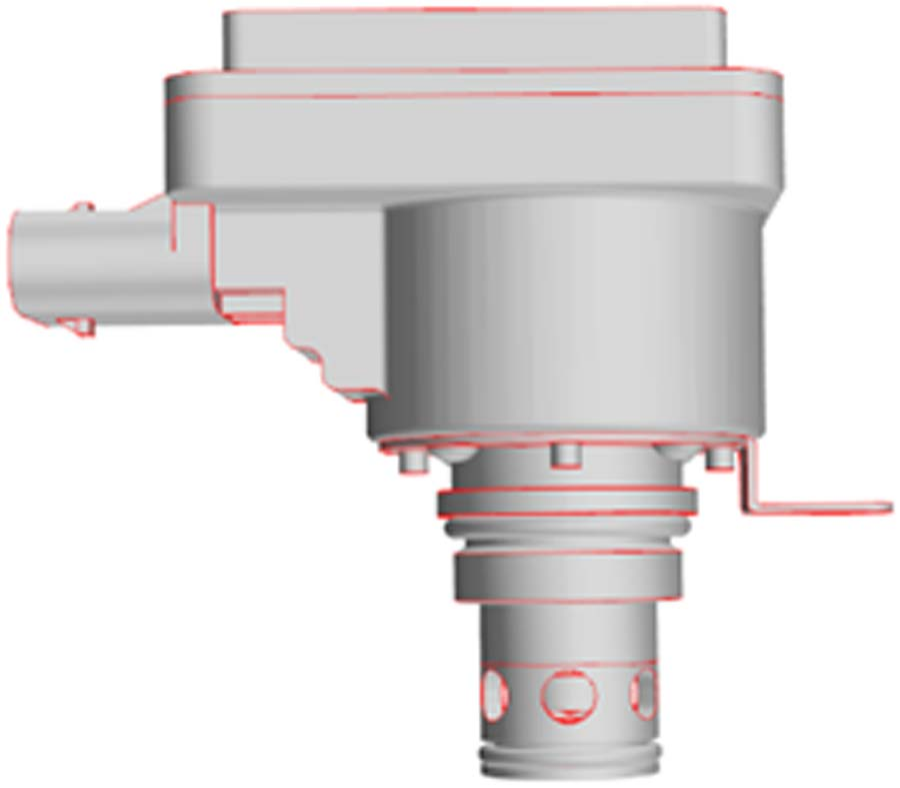
Three-dimensional model of electronic expansion valve.

The noise generation of electronic expansion valves is principally associated with their internal geometry and operating parameters. As demonstrated in Fig 1, the valve core is conically shaped, and its opening is meticulously regulated by the stem stroke. As the refrigerant flows through the annular slit between the spool and the valve seat, there is an abrupt decrease in the cross-sectional area of the flow. This results in a sharp increase in the flow rate from the inlet, from 2 m/s to 30 ~ 60 m/s. According to Bernoulli’s equation, the local pressure drops below the saturated vapor pressure (e.g., the outlet pressure is 0.8 MPa when the opening degree is 1.25 mm, which is lower than the saturated vapor pressure of R134a at 35°C, 0.887 MPa), which triggers flash cavitation. The collapse of the vacuum in the high-pressure area (i.e., the evaporator inlet) has been observed to occur in the presence of micro-jets and shock waves. The pressure pulsation amplitude of these phenomena has been recorded as up to ± 0.2 MPa. It has been demonstrated that the transmission of micro-jets and shock waves through the tube wall, in conjunction with tube wall vibration and sound radiation, results in the formation of broadband noise. The main frequency of this noise has been determined to be within the range of 800 and 2200 Hz.

In the electronic expansion valve, the refrigerant is converted from liquid to gas as it expands, creating a two-phase flow. The orifice at the pressure drop converts some of the pressure energy into kinetic energy, causing the liquid to flow out at high speed. When the pressure drops below the saturated vapor pressure, the distance between the refrigerant molecules increases and the interaction force is weakened, making it easier to evaporate into a gas. As the pressure decreases, the gases in the liquid precipitate bubbles, which come from dissolved gases or evaporation of the liquid. As the pressure continues to fall, the bubbles increase in number and size. As the refrigerant flows through the sudden expansion of the pipe, the pressure rises, causing the bubbles to burst, creating a shock wave and a large pressure pulsation that causes vibration and noise in the pipe [21]. This is the main part of the flow-induced noise.

Concurrently, due to the abrupt contraction of the flow channel, the fluid undergoes extrusion and collision, while also generating thermal and acoustic energy. In particular, acoustic energy is emitted from the throttle in the form of radiation, thereby generating noise [22]. It constitutes a secondary component of the flow-induced noise.

### 2.2 Electronic expansion valve physical model

**Fig 2:**
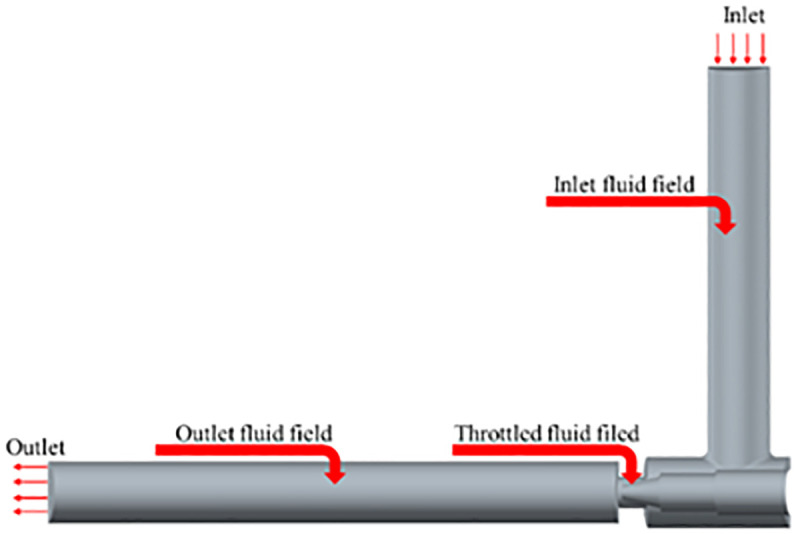
CFD fluid domain model of electronic expansion valve.

The three-dimensional model of the electronic expansion valve is shown in Fig 1. In the fluid simulation, the non-critical parts are simplified to improve the computational efficiency. The inlet and outlet are extended to keep the inlet flow rate uniform and avoid backflow interference, while the refrigerant pressure drop at the throttled fluid field represents a critical location in the flow path of an electronic expansion valve. Fig 2 shows the flow channel model extracted from Catia for flow field calculation.

### 2.3 Mathematical model

In this study, Star-CCM + 2302 is used for steady state simulations, the pressure-based coupling algorithm is selected for the solver, and the spatial discretization format is adopted as the second-order windward format to improve the computational accuracy. The turbulence model is the Realizable k-ε model, which can more accurately predict the turbulent kinetic energy dissipation rate of strong shear and rotating flows. The cavitation model is the Schneer-Sauer single-equation model, which describes the phase transition process by introducing the number density of vacuoles. This model has better convergence in high-speed cavitation flows than the Zwart-Gerber-Belamri model. The FW-H (Ffowcs Williams-Hawkings) equation is chosen for the acoustic model, which is used to calculate the far-field noise radiation by extracting the turbulent pulsation pressure in the fluid domain as the sound source term, combined with the free-field Green’s function [23].

#### 2.3.1 Fluid mechanics model.

Mass conservation equation as in (1)


∂ρ/∂t+∇·(ρU)=0 (1)

Energy conservation equation as in (2)


$$\partial (\rho T)/\partial t+\nabla \cdot (\rho UT)=\nabla \cdot (k/c_{p}\cdot gradT)+S_{T}$$ (2)

Momentum conservation equation as in (3)–(5)


$$\rho \frac{du}{dt}=\rho F_{bx}+\frac{\partial p_{xx}}{\partial x}+\frac{\partial p_{yx}}{\partial y}+\frac{\partial p_{zx}}{\partial z}$$ (3)

$$\rho \frac{dv}{dt}=\rho F_{by}+\frac{\partial p_{xy}}{\partial x}+\frac{\partial p_{yy}}{\partial y}+\frac{\partial p_{zy}}{\partial z}$$ (4)

$$\rho \frac{dw}{dt}=\rho F_{bz}+\frac{\partial p_{xz}}{\partial x}+\frac{\partial p_{yz}}{\partial y}+\frac{\partial p_{zz}}{\partial z}$$ (5)

ρ is the fluid density, kg/m^3^. t is the time, s. U is the fluid velocity vector, m/s. u is the vector of U in the x-direction, m/s. v is the vector of U in the y-direction, m/s. w is the vector of U in the z-direction, m/s. T is the temperature, K. k is the thermal conductivity of the fluid, W/(m·K). cp is the constant-pressure specific heat capacity, J/(kg·K). S_T_ is the internal heat source of the fluid and the portion of the fluid’s mechanical energy that is acted upon by the viscosity. F_bx_, F_by_, F_bz_ is the component of the mass force per unit of fluid in the x, y, and z directions, N. p_xx_, p_yx_, etc. are components of the fluid internal stress tensor, N.

#### 2.3.2 Cavitation model.

Cavitation models describe the formation of cavities by the evaporation of liquids under specific conditions. They are constructed by taking into account pressure changes, bubble formation and collapse, interphase mass transport and momentum exchange. Pressure and flow rate are the most critical, so they are used to define the cavitation number, which expresses the cavitation generation and state, and is given by (6) [24]:


$$\sigma_{k}=(p_{\infty }-p_{v})/\frac{1}{2}\rho v_{\infty }^{2}$$ (6)

v is the cross-sectional fluid flow rate, m/s. p is the cross-sectional fluid pressure, Pa. p_v_ is the saturated vapor pressure of the liquid, Pa. When the lowest pressure in the liquid flow field reaches the critical pressure p_i_ to stabilize the gas nucleus, cavitation occurs there, and the cavitation number in this state is the critical cavitation number, also called the incipient cavitation number σ_ki_:


$$\sigma_{ki}=(p_{i}-p_{v})/\frac{1}{2}\rho v_{\infty }^{2}$$ (7)

**Fig 3:**
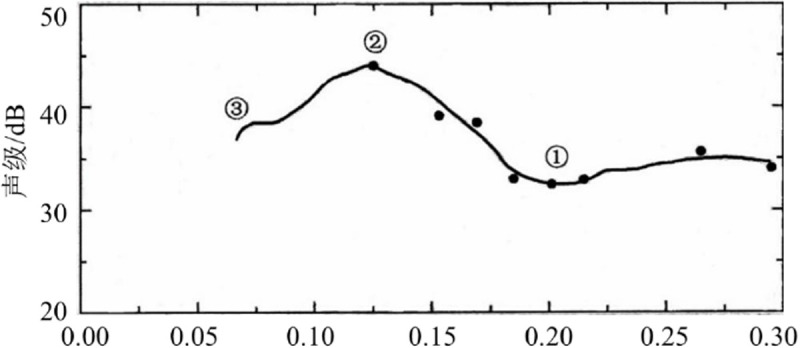
Variation of cavitation number vs. cavitation noise.

Cavitation does not occur when σ_k_ > σ_ki_. Cavitation occurs when σ_k _≤ σ_ki_. The number of vacuoles increases greatly, and the smaller the number of vacuoles, the greater the intensity of cavitation. When σ_k_ <<σ_ki_, cavitation is fully developed, exhibiting a supercavitation state. Arndt [25] found that when the fluid is in a super-cavitation state, the cavitation number is reduced to a certain degree, such as the②-③section in Fig 3, the sound pressure level of the cavitation noise does not increase but decreases.

The Schneer-Sauer cavitation model is simple and efficient, converges quickly and robustly compared to other models, and contains only one empirical constant, the vacuole number density. The governing equations are [26]:


$$p\leq p_{v},R_{e}=\frac{\rho_{v}\rho_{l}}{\rho }\alpha_{v}(1-\alpha_{v})\frac{3}{R_{B}}\sqrt{\frac{2(p_{v}-p)}{3\rho_{l}}}$$ (8)

$$p>p_{v},R_{c}=\frac{\rho_{v}\rho_{l}}{\rho }\alpha_{v}(1-\alpha_{v})\frac{3}{R_{B}}\sqrt{\frac{2(p-p_{v})}{3\rho_{l}}}$$ (9)

$$\begin{array}{c} R_{B}={\left(\frac{\alpha_{v}}{1-\alpha_{v}}\frac{3}{4\pi }\frac{1}{n}\right)}^{1/3} \end{array}$$ (10)

α_v_ is the gas-phase volume fraction. R_B_ is the radius of the vacuole, m. ρ_l_ is the density of the liquid, kg/m^3^. ρ_v_ is the density of the gas, kg/m^3^. R_e_ is the evaporative phase generation rate. R_c_ is the condensed phase generation rate. n is the vacuole number density, and n = 1 × 10^13^ is taken in the model.

#### 2.3.3 Acoustical model.

Lighthill derives the total turbulent noise power in a free jet as in


$$W=K\rho^{2}D^{2}v^{8}/\rho_{0}c_{0}^{5}$$ (11)

W is the turbulent total sound rate, W; D is the orifice aperture, m. v is the fluid velocity, m/s. ρ_0_ is the density of the sound propagation medium, kg/m^3^. c_0_ is the speed of sound, m/s. K is the Lighthill coefficient and experimental value is approximately 0.3 × 10^−4^ ~ 1.8 × 10^−4^.

The sound power per unit volume is


$$P_{A}=\alpha_{\varepsilon }\rho_{0}\varepsilon M_{t}^{5}$$ (12)

$$M_{t}=\sqrt{2k_{T}}/a_{0}$$ (13)

The sound power level L_P_ is


$$L_{P}=10\mathrm{lg}(P_{A}/P_{ref})$$ (14)

α_ε_ is a constant with a value of 0.1. ε is the turbulent dissipation rate. k_T_ is the turbulent kinetic energy, J. a_0_ is the local speed of sound, m/s. α is a constant. P_ref_ is the reference sound power, W.

#### 2.3.4 Model assumptions and limitations.

(1) Schneer-Sauer cavitation model

The Schneer-Sauer cavitation model is predicated on the following fundamental assumptions: Firstly, it is assumed that the bubbles are uniformly distributed in the fluid and of a single size. This assumption disregards the aggregation and fragmentation behavior of bubbles and the polydispersity characteristics that may be present in the actual working conditions. Secondly, the phase transition process is regarded as a proposed steady state. The pressure-driven evaporation and condensation are only considered, and the influences of temperature gradient and inertia effects on the phase transition rate are not included. Finally, the model defaults to no interaction between bubbles and is applicable to low gas content scenarios. However, the model is not without its limitations, namely that its prediction accuracy may be significantly reduced when simulating high gas content or transient cavitation (e.g., shock waves caused by bubble collapse).

(2) FW-H acoustic radiation model

The FW-H acoustic radiation model is constructed on the basis of linear acoustic propagation theory. This theory assumes that nonlinear effects (e.g., waveform distortion at high sound pressure) are negligible during the propagation of the acoustic field. Furthermore, it assumes that the acoustic source is generated exclusively by hydrodynamic fluctuations (turbulence and cavitation) and that the coupling between solid-boundary vibrations and the acoustic field is not accounted for. Concurrently, the model operates under the assumption that the sound velocity is uniformly distributed within the computational domain. It does not, however, take into account the effects of temperature gradient and flow velocity on the sound propagation path. The model is limited in two ways. Firstly, the prediction of low-frequency noise (<500 Hz) may be inaccurate due to the exclusion of the resonance effect of the pipeline structure. Secondly, the attenuation characteristics of high-frequency noise (>5 kHz) may be underestimated because the model does not take into account the influence of the material’s sound.

## 3 Simulation analysis of flow field

### 3.1 Mesh model validation

**Fig 4:**
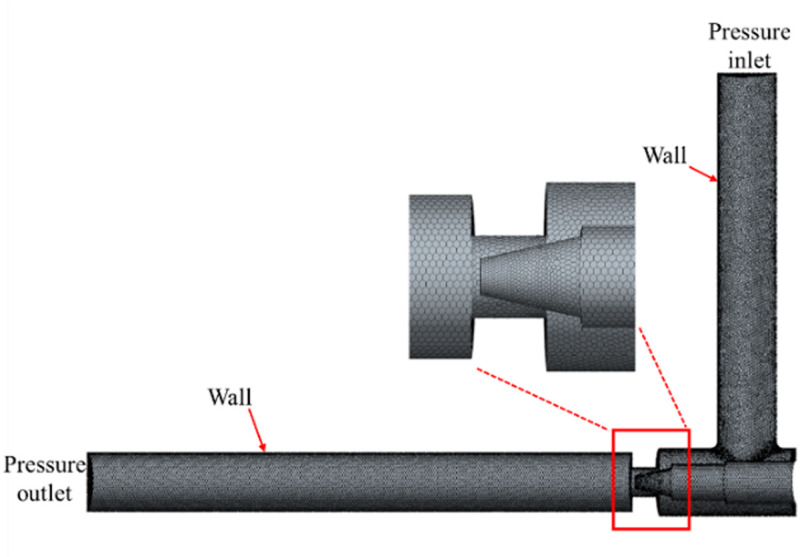
Mesh delineation of the fluid computational domain.

**Fig 5:**
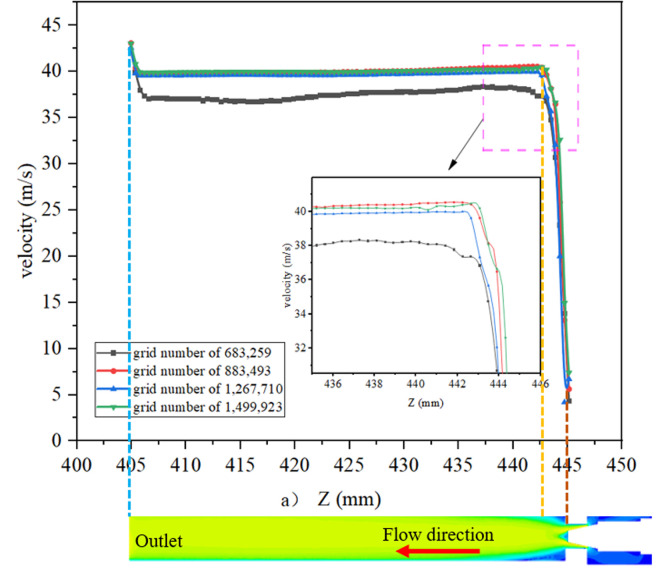
Verification of velocity change in Z-direction.

**Fig 6:**
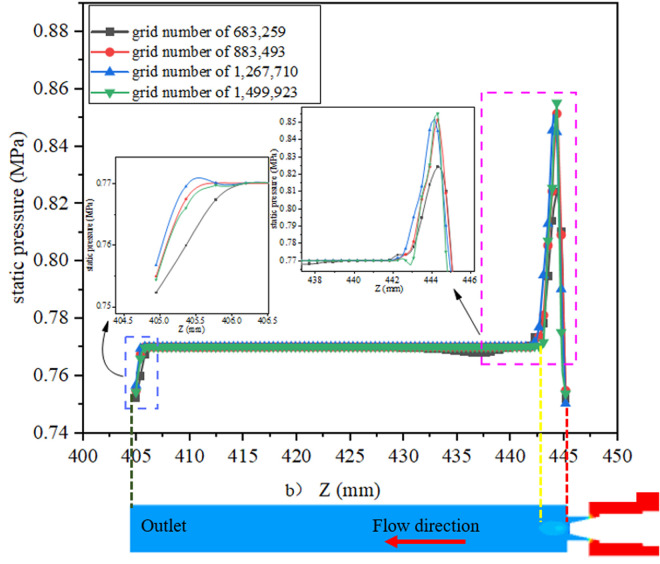
Verification of static pressure change in Z-direction.

The flow field is analyzed using Star CCM + , and the flow field at the valve needle is complex with large velocity and pressure gradients, so the mesh is coded there. Fig 4 shows the model fluid domain mesh division. Mesh sensitivity is simulated by CFD to determine the appropriate mesh size to improve computational accuracy and stability. Fig 5–6 and Table 1 show the relationship between the number of meshes and the error with velocity and pressure as reference and the outlet mass flow, respectively. The results show that with 0.25 mm grid size and 883,000 mesh number, the velocity and pressure curves are well fitted and the error of the outlet flow rate is small, so this grid model is selected for CFD analysis [27].

**Table 1:** Grid independence test.

Mesh size	Grid number / ten thousand	Mass flow rate /(kg/s)	Error
0.3	68.3	0. 116	4.92%
0.25	88.3	0.120	1.64%
0.2	126.8	0.121	0.82%
0.18	150	0.122	0

### 3.2 Boundary condition determination

The boundary conditions are established in accordance with the prevailing MAC operating conditions. The inlet is designated as a pressure inlet, with the total pressure value being set in accordance with the respective operating conditions of varying pulse numbers as outlined in Table 2 (1.5–3.7 MPa). The inlet turbulence intensity is set at 5%, and the hydrodynamic diameter is designated as the equivalent diameter of the valve body, set at 5 mm. The outlet is configured as a pressure outlet, with the static pressure value being set in accordance with the back pressure of the evaporator (0.71–1.2 MPa). The wall surface is subject to no-slip boundary conditions, and the material of the valve body is set as brass in the coupled heat transfer calculation (thermal conductivity 109 W/(m-K)). The convective heat transfer coefficient with the environment is 15 W/(m^2^-K). The temporal resolution of the system was set at 0.001 seconds, and the convergence criteria stipulated that the residuals of the equations were to be reduced to below 10 ⁻ ⁴, and that the standard deviation of the parameters at the monitoring points was to be less than 1%.

**Table 2:** Relationship between the number of pulses and the degree of opening.

Pulse number	Opening degree (mm)	Inlet pressure (MPa)	Outlet pressure (MPa)	Inlet temperature (°C)
100	0.625	1.5	0.71	25
150	0.9375	2.2	0.75	30
200	1.25	2.75	0.8	35
250	1.5625	2.95	0.865	40
300	1.875	3.2	0.95	45
350	2.1875	3.7	1.2	50

The state of the R134a refrigerant varies according to the operating conditions and its physical parameters also vary according to the temperature. The opening of the electronic expansion valve is determined by the rising height of the valve stem, which by default is 0. Different pulse numbers are used as a reference to correspond to different openings, physical parameters and operating conditions [28], as shown in Table 2 and Table 3.

**Table 3:** Physical properties of refrigerants under different operating conditions.

Opening degree(mm)	Inlet temperature(°C)	liquid density (kg/m^3^)	dynamic viscosity (Pa·s)	gas density (kg/m^3^)	Saturated pressure (Pa)
0.625	25	1206.71	1.955e-4	32.35	665400
0.9375	30	1187.46	1.923e-4	37.535	770200
1.25	35	1167.50	1.716e-4	43.416	887000
1.5625	40	1146.74	1.617e-4	50.085	1016600
1.875	45	1125.05	1.519e-4	57.657	1159900
2.1875	50	1102.31	1.422e-4	66.272	1317900

### 3.3 Flow field analysis

To analyze the cavitation characteristics under different working conditions, simulation under the six operating conditions in Table 2 are taken as an example. Results show the larger the opening degree represents the higher the ambient temperature inside the vehicle under the operating condition, which needs to reduce the temperature as soon as possible, and the required refrigerant flow rate is larger. The distributions of its pressure field, velocity field, gas phase volume fraction, turbulence kinetic energy and noise are simulated.

**Fig 7:**
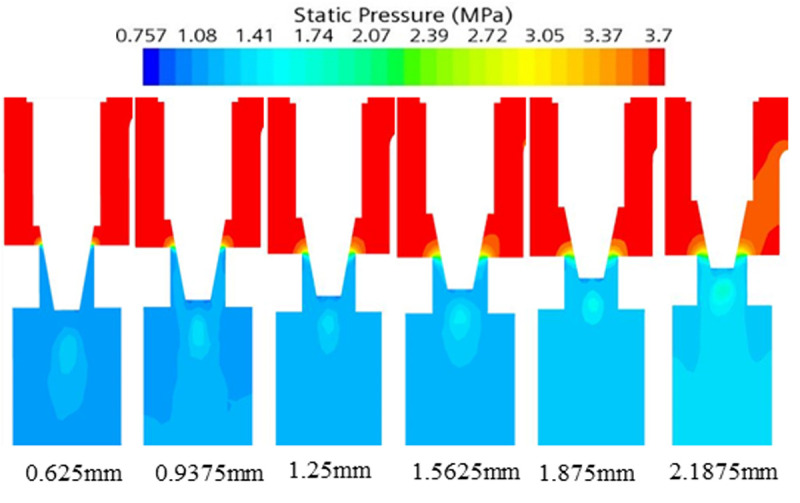
Pressure cloud of electronic expansion valve under various operating conditions.

**Fig 8:**
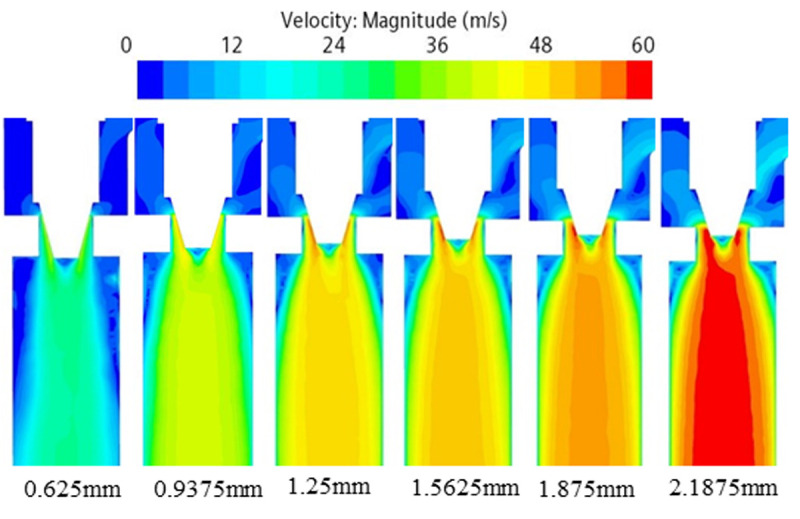
Velocity cloud of electronic expansion valve under various operating conditions.

**Fig 9:**
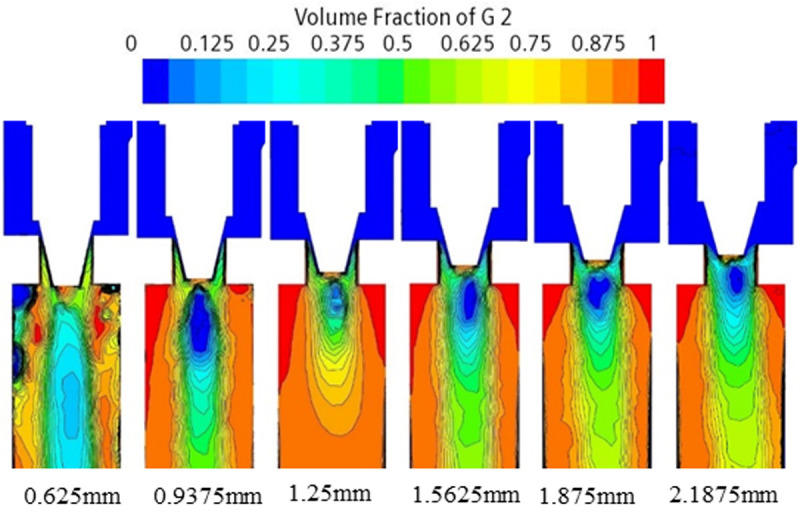
Gas phase volume fraction cloud of electronic expansion valve under various operating conditions.

Through the pressure, velocity and gas phase volume fraction distribution of cloud diagrams from Figs 7–9 can be obtained. Flow field distribution trends under the various orifices are generally similar. The pressure at the inlet is a high-pressure state. But upon passing through the valve core of the expansion valve, the pressure is reduced from 1.5MPa ~ 3.7MPa to 0.6MPa, with some of the energy being converted to kinetic energy. The refrigerant flow rate increases rapidly from 2 m/s at the inlet to 30 m/s ~ 60 m/s in the form of a jet flow. The liquid is primarily concentrated in the valve body’s central region, while the gas is distributed along the valve body’s wall. The liquid flow rate is comparatively higher than that of the gas. The ratio of the volume of the gas phase to the volume of the gas-liquid mixed phase is employed as an index for evaluating the severity of cavitation at the location in discussion. The average vapor volume fraction, which indicates the ratio of the total vapor volume in the fluid domain to the total fluid domain volume, is increased from 0 at the inlet to 50% or even higher. As the opening increases, the pressure drop and refrigerant flow rate at the throttle gradually increase. The pressure decreases, the pressure less than the saturation vapor pressure increases in the region, the formation of vapor bubbles increases, the average vapor volume fraction increases, and the cavitation phenomenon is enhanced.

**Fig 10:**
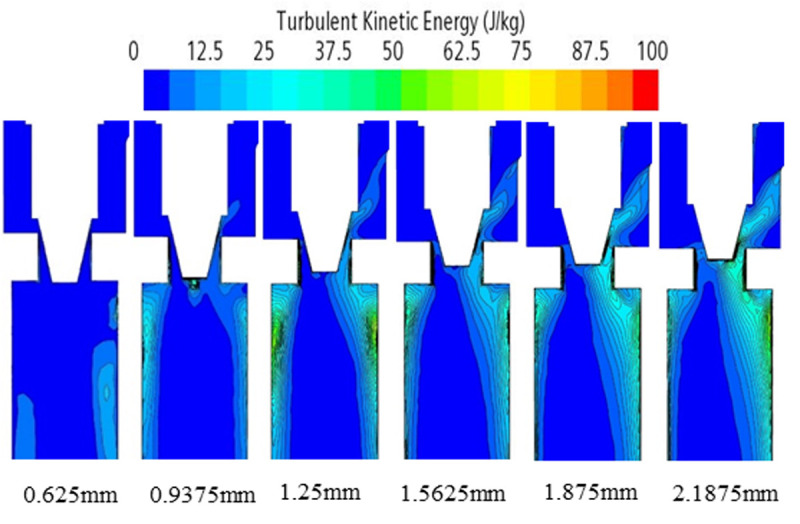
Turbulent energy distribution of electronic expansion valve under various operating conditions.

**Fig 11:**
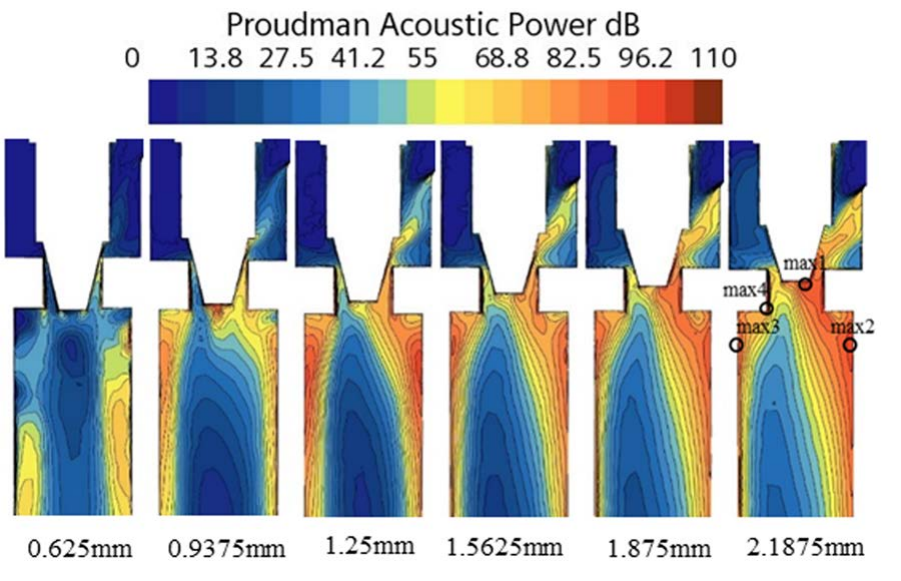
Turbulent energy distribution of electronic expansion valve under various operating conditions.

According to the analysis of the cloud diagrams in Figs 7–9, when the opening of the electronic expansion valve was increased from 0.625 mm to 2.1875 mm, the volume of the low-pressure region downstream of the valve spool (p < 0.5 MPa) was enlarged by 37%, and the mean value of the gas-phase volume fraction increased from 42% to 58%. Concurrently, the vacuole collapse frequency was shifted from 1200 Hz to the high-frequency band (>2000 Hz), resulting in the broadening of the noise spectrum. When considered in conjunction with the theoretical model of equations (12)–(14), it becomes evident that the turbulent kinetic energy density is quadratic in relation to the sound power. Furthermore, the peak turbulent kinetic energy in the spool wake area attains a magnitude of 28 m^2^/s^2^ when the opening degree is elevated to 2.1875 mm. This represents a 2.4-fold increase in comparison to the baseline condition (opening degree of 0.625 mm) and is concomitant with an augmentation of the sound pressure level of 9.6dB. Furthermore, a comparison of the turbulence kinetic energy distribution with the quadrupole noise map in Figs 10–11 reveals a correlation between the location of the noise source and the high frequency of the noise source. The findings indicate a strong correlation between the location of the noise source and the region of high turbulent kinetic energy. Furthermore, it is observed that the turbulent kinetic energy below the spool exhibits a substantial increase when the orifice opening is augmented. This finding suggests that the refrigerant flow noise is predominantly characterized by turbulent kinetic energy, and its spatial distribution and intensity can be reliably predicted by the turbulent kinetic energy parameter [29].

### 3.4 Relationship between thermodynamic and acoustic parameters

Changes in thermodynamic parameters affect refrigerant properties and flow characteristics, causing noise through mechanisms such as cavitation and pressure pulsation. When the high temperature in the car needs to be urgently cooled, the opening of the electronic expansion valve is increased and the flow of refrigerant is increased, resulting in a rapid local temperature rise and pressure increase, and the dynamic viscosity is reduced. The fluid temperature rises, molecular thermal motion increases, and the collision energy loss at the valve core and pipe wall increases, which is partially converted into acoustic energy to produce noise.

According to Bernoulli’s equation, the refrigerant flow rate increases dramatically as it flows through the valve core, and the pressure decreases due to the constriction of the flow path. After entering the evaporator, the tube diameter expands, causing the pressure to rise. Meanwhile, cavitation bubbles burst in the high pressure region, creating shock waves and triggering large pressure pulsations that cause vibration and noise in the tube. According to the analysis of Eq. 12–14 and Fig 7–11, it is clear that there is a positive relationship between noise and turbulence energy. As the refrigerant flow increases, the turbulence energy in the valve also increases, which makes the fluid flow become more unstable, resulting in a significant increase in the noise sound pressure level.

## 4 Optimization of vapor-doped supercavitation

According to the gas phase distribution in Fig 9, the gas phase is concentrated in the wall of the valve body. In order to inhibit cavitation, it is proposed to use a bypass tube to guide the refrigerant gas to the outlet of the electronic expansion valve by using the pressure difference between the evaporator inlet and the outlet of the electronic expansion valve to increase the local gas content and form supercavitation, which increases the pressure and reduces the number of cavitations, thus reducing cavitation bubbles and noise.

### 4.1 Vapor-doped supercavitation flow field analysis

**Fig 12:**
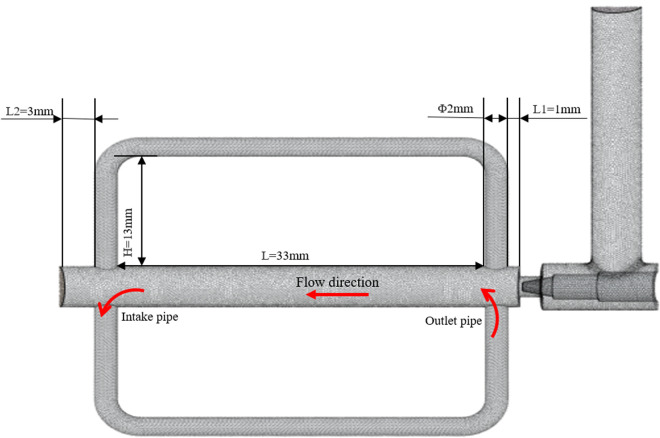
Dual bypass pipe model.

**Fig 13:**
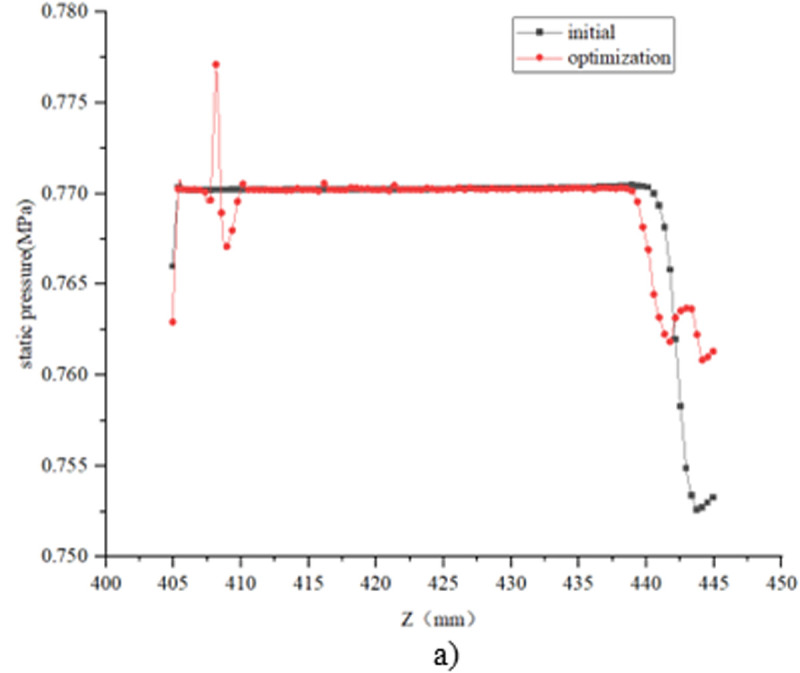
Change in static pressure along the Z-direction with the addition of a bypass tube.

**Fig 14:**
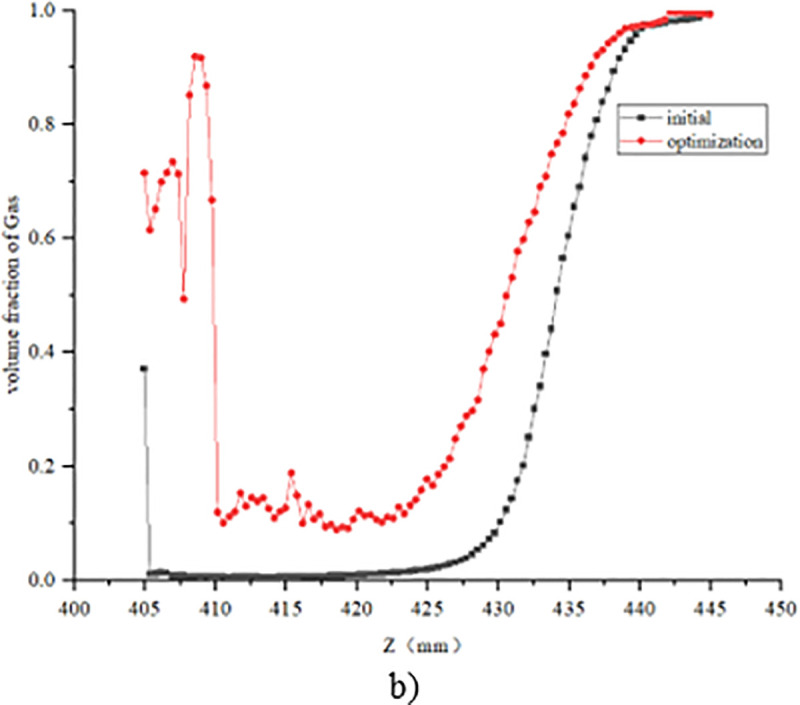
Change in gas phase volume fraction along the Z direction with the addition of a bypass tube.

**Fig 15:**
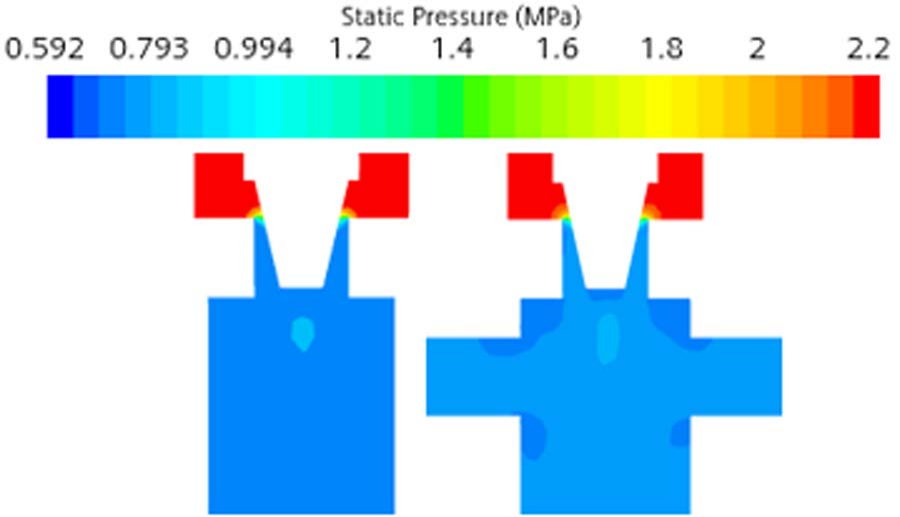
Comparison of pressure before and after optimization cloud.

The structural optimization of the bypass pipe is proposed, and the bypass pipe includes the inlet pipe and the outlet pipe, as shown in Fig 12 When the liquid refrigerant flows through the electronic expansion valve into a gas-liquid mixture, the liquid along the center of the pipeline flows like a jet, and gas seeps into the bypass pipe intake pipe along the pipe wall. When the internal flow field gradually stabilized, the pressure at the outlet pipe is lower than that at the intake pipe, and the gas flows from the intake pipe into the bypass pipe, and out to the outlet pipe, increasing the gas content and pressure of the outlet of the electronic expansion valve. Taking the case of pulse number 150 as an example, the comparison of changes in pressure and gas phase volume fraction before and after steam-doped optimization is shown in Figs 13–15. It can be seen that after vapor doping, the gas phase volume fraction at the outlet increases. The pressure at the smallest point increases from 0.751 MPa to 0.762 MPa with an increase of 1.5%. The total turbulence kinetic energy decreases, the vortex scale decreases, and the number of cavitation nuclei and bubbles in the cavitation primordium decreases, which suppresses the generation of the cavitation, resulting in the noise reduction.

### 4.2 Physical mechanism of noise reduction by vapor-doped supercavitation

#### 4.2.1 Vapor phase thickening and pressure compensation.

The refrigerant vapor through the bypass tube significantly increased the gas phase volume fraction at the valve outlet (from 58% to 72%). The saturated vapor pressure of the mixed fluid increased after vapor-doped, and the local pressure increased by about 1.5% (from 0.751 MPa to 0.762 MPa), which was higher than the saturated vapor pressure threshold of the refrigerant, and thus suppressed the incipient cavitation. CFD simulations showed that the number of cavitations was increased above the threshold and the number of cavitations was reduced by 63%.

#### 4.2.2 Energy dissipation in vacuolar extinction.

Vapor-doped prolongs the collapse time of the vacuole by increasing the ambient pressure and reduces the peak collapse shock pressure by 32%. At the same time, vapor-doped reduces the wall asymmetry of the vacuole, reduces the microjet velocity by 46 per cent, and increases the energy dissipation rate by 65 per cent, effectively weakening the intensity of the shock wave.

### 4.3 Noise changes at the observation point

**Fig 16:**
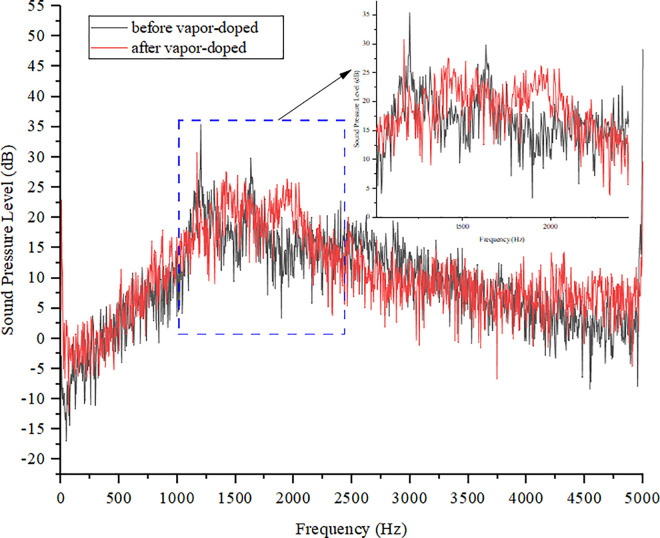
Far-field noise sound pressure level before and after vapor-doped.

In order to accurately analyze the far-field noise characteristics, four monitoring points were set up, and the positions were set according to the noise maximum points in Fig 11, which were 0.5 m away from the objects to be measured [30]. Fig 16 shows that the peak of the sound pressure level after vapor-doped was concentrated in the range of 1000–2200 Hz, and the maximum value was reduced to 30.72 dB at 1167.55 Hz, which was 4.69 dB lower than that before vapor-doped, which verifies the effectiveness of the strategy of vapor-doped supercavitation.

### 4.4 Basis for selection of structural parameters of vapor-doped components

The optimal selection of the structural parameters of the vapor-doped components is based on the following multidimensional criteria:

(1) Hydrodynamic constraints on the diameter of the vapor-doped pipe

The simulation results show that insufficient vapor-doped (<0.8% doping rate) with too small a diameter (Φ < 1 mm) results in weak noise reduction (<1 dB reduction in SPL), while too large a diameter (Φ > 2.5 mm) triggers a high-speed vapor-doped flow (>15 m/s), which generates additional vortex noise (1.8 dB increase in SPL in the 500–800 Hz frequency band). The equilibrium point was determined to be Φ = 2 mm, at which the doping rate reaches 1.9%, the cavitation number is increased above the critical value, and the main frequency noise is reduced by 4.2 dB.

(2) Flow field matching mechanism of vapor-doped pipe angle

When the angle α = 35°, the dopant flow forms an 18° angle with the main flow, delaying the separation point of the gas-liquid shear layer to 5 mm downstream of the valve, and improving the uniformity of doping by 23%.

(3) Energy dissipation regulation of vapor-doped pipe height

At a height of 15 mm, the pressure drop in the doped gas pipe is 0.03 MPa (compared to 0.12 MPa at a height of 10 mm). This reduction in pressure leads to a decrease in the kinetic energy loss within the gas phase, thereby stabilizing the vapor-doped flow rate at a range of 12–14 m/s.

### 4.5 Effect of vapor-doped structure on cavitation

Aiming at the impact of the structural design and arrangement of the vapor-doped components on the flow field, the following key structural parameters are extracted, corresponding to the model in Fig 12, and the main structural parameters of the bypass pipe are shown in Table 4. Since the formation of the vapor-doped supercavitation flow field requires the bypass pipe to introduce the gas at the intake pipe to the outlet pipe near the spool of the electronic expansion valve, the positions of the inlet and outlet of the bypass pipe need to be fixed, this means the values of L, L1, and L2 are fixed to analyze the influence of the remaining three structural parameters.

**Table 4:** Main structural parameters of bypass pipe.

structural parameters	values
Bypass pipe length L (mm)	33
Distance from bypass tube to valve core L1(mm)	1
Distance from bypass pipe to EXV outlet L2(mm)	3
Bypass pipe height H(mm)	13
Bypass pipe diameter Φ(mm)	2
Bypass inlet pipe angle α(°)	0

Bypass pipe height H. The height of the bypass pipe affects not only the transmission distance of the gas, but also the flow state of the gas. The larger height will increase the flow resistance, while giving the gas more time and space for energy conversion and mixing. The smaller height will lead to just inflow of gas impact on the pipe wall, resulting in vibration and noise.

Bypass pipe diameter Φ. The diameter of the bypass pipe directly affects the gas flow capacity. The larger diameter can reduce the resistance of gas flow and increase the flow rate, which may affect the effect of vapor doping and the formation speed and quality of supercavitation flow field.

Bypass inlet pipe angle α. The angle of the bypass inlet pipe will affect the gas flow direction and velocity distribution. The right angle will make it easier for the gas to flow into the bypass pipe and reduce the pipeline resistance, but a larger bending angle will increase the flow resistance at the pipeline turn, and even cause eddy currents and turbulence, thus affecting the effect of vapor doping and the stability of the flow field [31].

## 5 Response surface optimization of vapor-doped components

**Fig 17:**
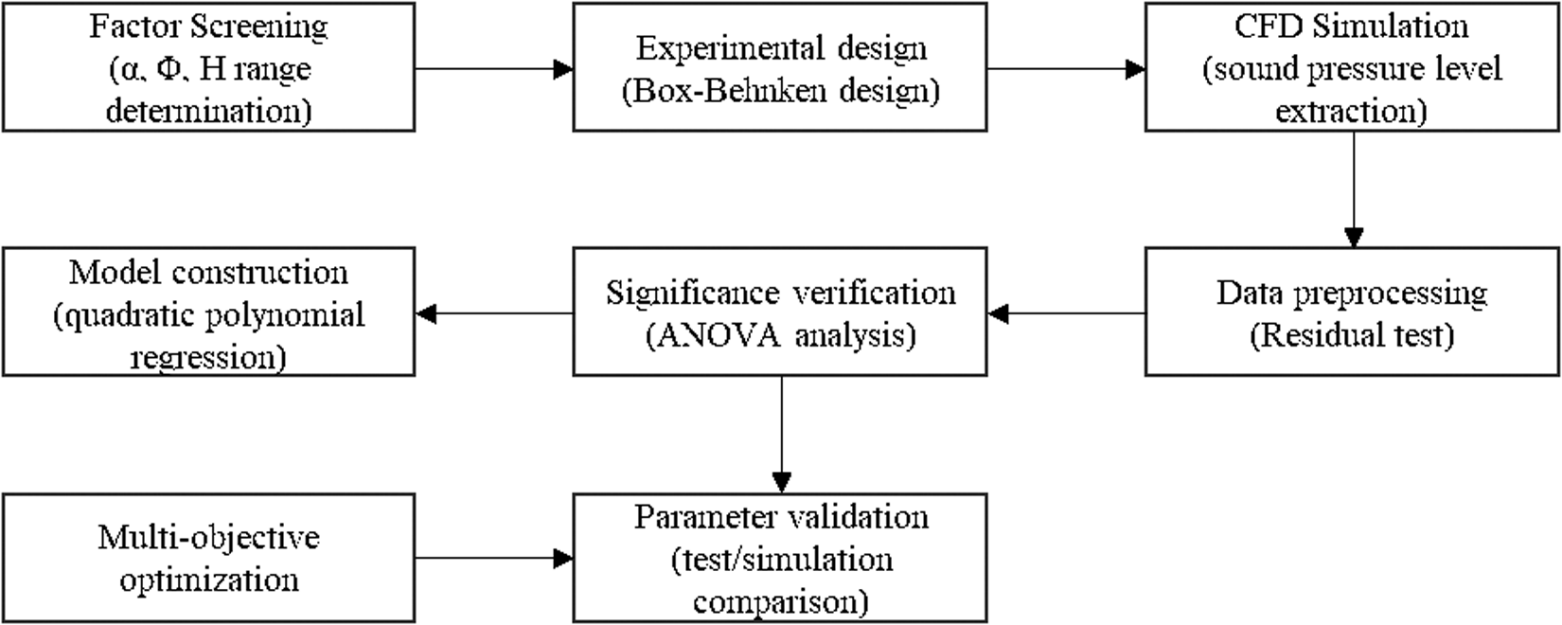
Optimization Flowchart.

Response surface optimization method uses experimental design and statistical analysis to construct an approximate model to replace a complex system with a finite number of experiments, and expresses the relationship between the response and the factors through a mathematical model to solve the optimal solution. The Box-Behnken method is simple in design, easy to interpret, and suitable for multi-factor interaction and multi-response optimization. In this study, the key parameters H, Φ and α are extracted for the vapor-doped component structure, and the response surface optimization is carried out with the peak sound pressure level and RMS as the response. The optimization process is shown in Fig 17.

### 5.1 Single-factor experiment

In this study, the Box-Behnken experimental design (BBD) was used for parameter optimization, which can reduce the number of trials by 33% compared with the central composite design (17 groups vs. 27 groups), and significantly improve the efficiency while ensuring the prediction accuracy. The following parameter intervals were determined through pre-tests and flow field simulation analysis: α was 20° ~ 50°, Φ was 1 ~ 3 mm, and H was 10 ~ 25 mm. The control variable method was used to adjust each parameter to vary within the set ranges to study the effects of single factors on the sound pressure level.

**Fig 18:**
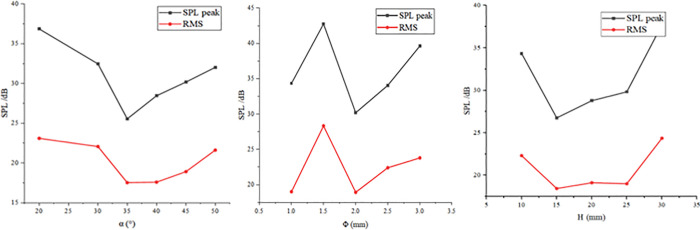
Results of single-factor experiment on key indicator parameters.

Based on the sensitivity analysis results of the one-factor test in Fig 18, a three-factor, three-level Box-Behnken design was used to construct the response surface optimization model in this study. The test levels of each parameter were set as follows: the optimization interval of α was determined to be 30°-40° (step size 5°), which can cover the confidence interval of the lowest point of sound pressure level (α = 35° ± 5°) in the one-factor test. The optimization interval of Φ was set to be 1.5–2.5 mm (step size 0.5 mm), which corresponds to the nonlinear response segment of flux sensitivity interval. The optimization of H was selected to be 10–20 mm (step size 5 mm), which can effectively balance the gas-phase transport efficiency with the antagonistic effect of wall impact.

### 5.2 Response surface methodology optimization

#### 5.2.1 Response surface experimental design.

Based on the results of single-factor experiment and the principle of Box-Behnken experiment [32], three factors and three levels were designed as shown in Table 5.

**Table 5:** Response surface analysis factors and levels.

Factors	Levels		
−1	0	1
α/°	30	35	40
Φ/mm	1.5	2	2.5
H/mm	10	15	20

Response surface optimization simulation experiments were carried out using peak sound pressure level and effective sound pressure level as response values and α, Φ and H as independent variables, as shown in Table 6.

**Table 6:** Response surface analysis factors and levels.

Experiment number	α/°	Φ/mm	H/mm	SPL-peak/dB	RMS/dB
1	40	2	20	30.56	24.95
2	35	2.5	10	35.6	21.34
3	35	1.5	10	34.26	23.18
4	35	2	15	25.56	17.55
5	30	1.5	15	30.77	22.65
6	35	2	15	25.56	17.55
7	40	2	10	36.99	23.87
8	40	2.5	15	31.18	24.63
9	30	2.5	15	34.60	24.43
10	40	1.5	15	30.76	23.07
11	35	1.5	20	32.62	22.43
12	35	2	15	25.56	17.55
13	35	2	15	25.56	17.55
14	35	2	15	25.56	17.55
15	30	2	20	34.02	25.54
16	30	2	10	34.97	22.47
17	35	2.5	20	34.63	24.58

#### 5.2.2 ANOVA results.

Response surface equations for peak sound pressure level (SPL peak) and effective sound pressure level (RMS) were constructed by a quadratic polynomial regression model with linear, interaction and quadratic terms, which was verified by analysis of variance (ANOVA) to be highly significant (p < 0.0001), as shown in equations (15) and (16). The ANOVA test shown in Table 7 indicates that the F-value indicates the significance of the overall fitted equation, with a large value indicating that the overall regression equation is highly significant. The P-value is used to determine whether a coefficient in the model or model is significant or not, and P < 0.05 in this regression model indicates that the difference in the model is extremely significant.

**Table 7:** ANOVA test of response function.

Source	Sum of Squares	df	Mean Square	F-value	p-value
Model	265.99	9	29.55	60.13	<0.0001
α	2.96	1	2.96	6.03	0.0437
Φ	7.22	1	7.22	14.69	0.0064
H	12.48	1	12.48	25.38	0.0015
αΦ	2.91	1	2.91	5.91	0.0453
αH	7.51	1	7.51	15.28	0.0058
ΦH	0.1122	1	0.1122	0.2283	0.6473
α^2^	39.49	1	39.49	80.35	<0.0001
Φ^2^	43.25	1	43.25	88.00	<0.0001
H^2^	127.95	1	127.95	260.33	<0.0001
Residual	3.44	7	0.4915		
Lack of Fit	3.44	3	1.15		
Pure Error	0.0000	4	0.0000		
Cor Total	269.43	16			
R^2^	0.9872				
Adjusted R^2^	0.9708				
Predicted R^2^	0.7957				

#### 5.2.3 Goodness-of-fit analysis.

R2 is the coefficient of determination, the larger it is the better the fit of the model. Adjusted R2 = 0.9708 indicates that 97.08% of the experimental data is available for this fitting equation. The difference between Predicted R^2^ = 0.7957 and Adjusted R^2^ is less than 0.2 indicating that the model has a good generalization between the training dataset and the test dataset.


$$\begin{array}{cc} SPL\left(dB\right)=\; & 230.0925-7.19275\alpha -38.45\Phi -5.08075H-0.341\alpha \Phi -0.0548\alpha H+0.067\Phi H\\ & +0.1225\alpha^{2}+12.82\Phi^{2}+0.2205H^{2} \end{array}$$ (15)

$$\begin{array}{cc} RMS\left(dB\right)=\; & 259.84375-10.07975\alpha -42.8625\Phi -3.4425H-0.022\alpha \Phi -0.0199\alpha H+0.399\Phi H\\ & +0.1494\alpha^{2}+9.64\Phi^{2}+0.1169H^{2} \end{array}$$ (16)

#### 5.2.4 Residual analysis.

**Fig 19:**
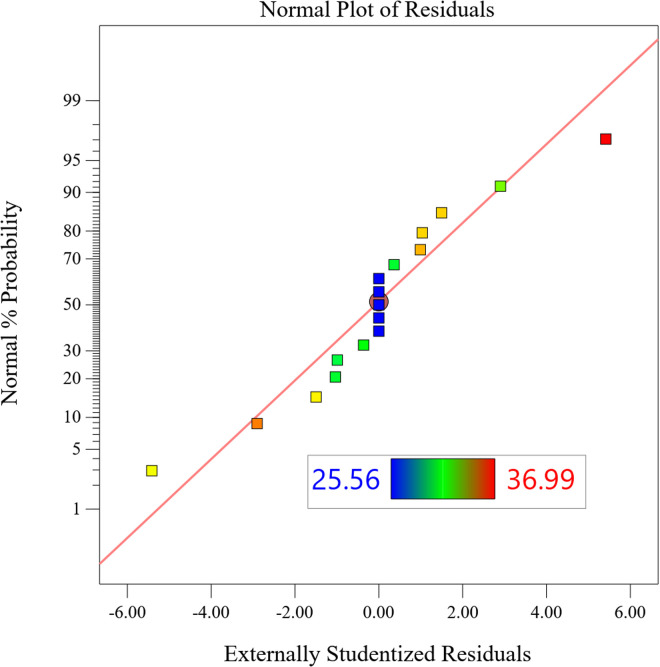
Normal probability plot.

**Fig 20:**
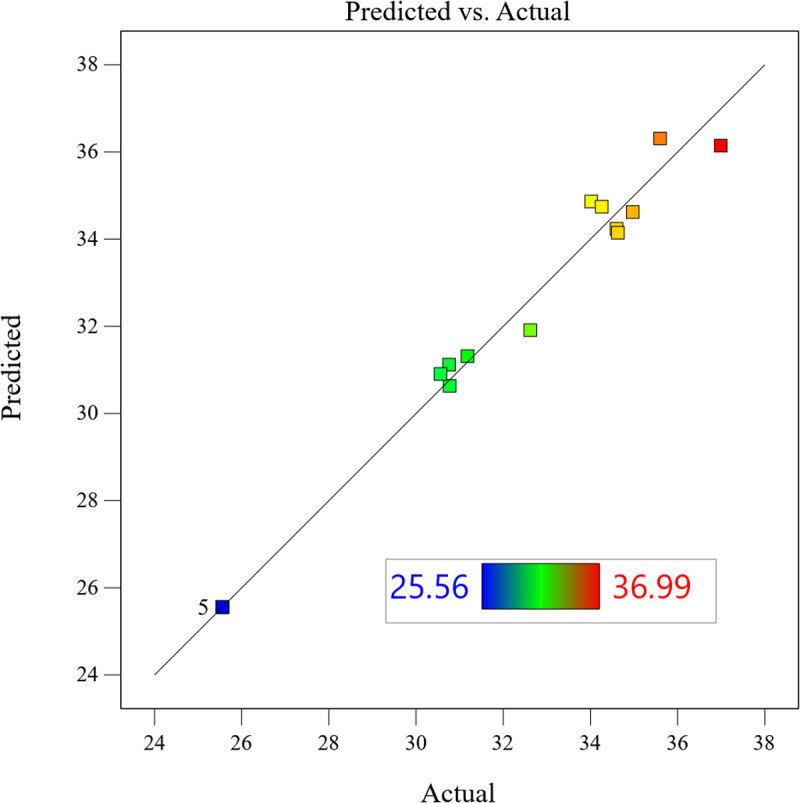
Response surface prediction model.

Fig 19 normal probability plot shows the relationship between the residuals and SPL peak, and the residual data points are distributed along the reference straight line, indicating that the model error obeys a normal distribution and satisfies the basic assumption of linear regression. The scatter of the actual and predicted values of SPL in Fig 20 shows a linear aggregation characteristic (slope k = 0.981), with a maximum absolute deviation of 1.2 dB and a root-mean-square error of 0.48 dB, which verifies the prediction reliability of the quadratic regression model.

The above analysis confirms that the constructed quadratic regression model is able to accurately characterize the influence law of multi-parameter interaction on the sound pressure level, and provides a high-confidence mathematical tool for the optimization of cavitation flow field.

#### 5.2.5 Response surface analysis.

**Fig 21:**
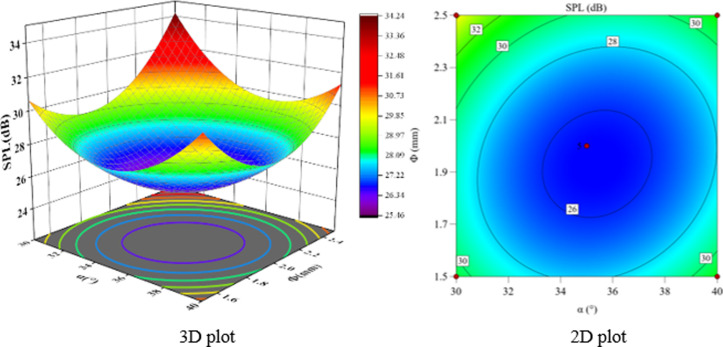
α and Φ vs. SPL.

**Fig 22:**
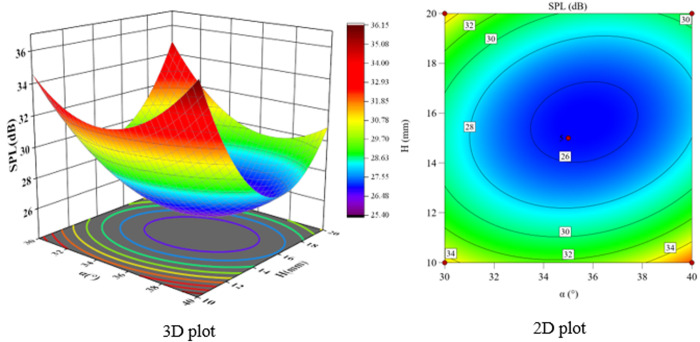
α and H vs. SPL.

**Fig 23:**
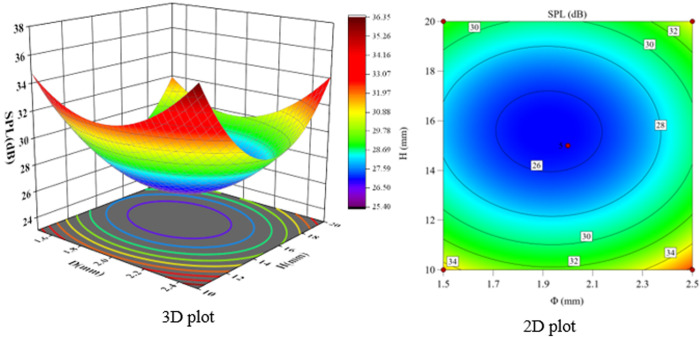
Φ and H vs. SPL.

**Fig 24:**
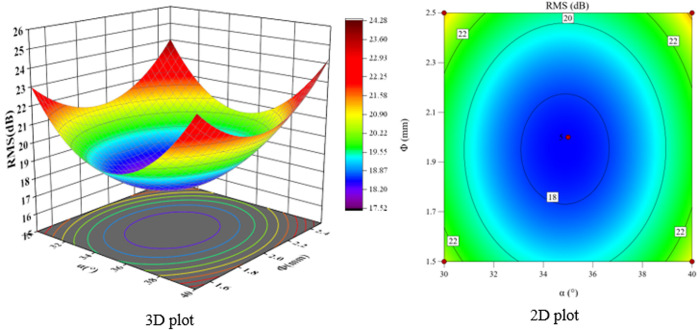
α and Φ vs. RMS.

**Fig 25:**
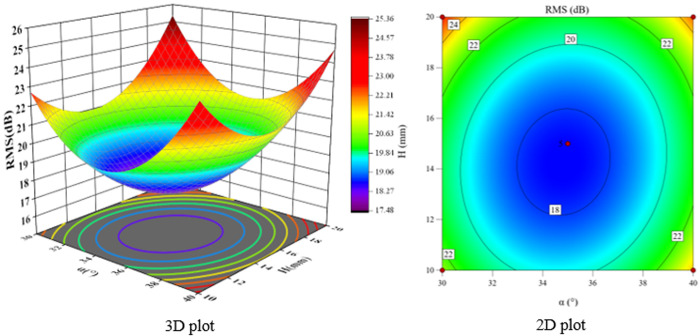
α and H vs. RMS.

**Fig 26:**
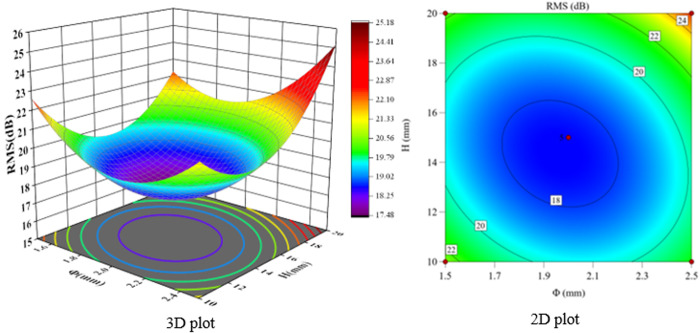
Φ and H vs. RMS.

According to the F-value and P-value tests, the order of the main factors affecting the peak sound pressure level SPL and the effective sound pressure level RMS is H > Φ > α. The experimental model was analyzed using Design-Expert 13, and three-dimensional and two-dimensional response surface plots were used to show the effects of the three-factor interactions of α, Φ, and H on the SPL and RMS, which are shown in Fig 21-26.

The interaction between fixing one factor at the center plane and the remaining two factors is examined in turn. Fig 21 and Fig 24 show the interactive effects of α and Φ when H is fixed at 15 mm. When α is 30°, SPL and RMS decrease and then increase with increasing Φ, with the inflection point occurring at approximately Φ = 2 mm. As α increases, both SPL and RMS decrease and then increase with Φ, and the location of the inflection point remains unchanged. The reason for this pattern is that as Φ increases, the amount of gas introduced to the spool outlet through the bypass tube increases, forming a supercavitation phenomenon, which increases the local pressure, reduces the number of cavitations, suppresses the generation of cavitation bubbles, and reduces the noise. If Φ is too large, the noise generated by the turbulent vortex caused by the bypass pipe at the inlet pipe is larger than the noise reduced by the formation of the supercavitation phenomenon, and therefore the sound pressure level starts to increase after a certain degree of reduction.

The response surface curves for the interactive effects of α and H with Φ fixed at 2 mm are shown in Fig 22 and Fig 25. The point of minimum value occurs near α = 35° and H = 15 mm. When α is 30°, H increases from 10 mm to 15 mm, SPL decreases from 34.97 dB to 29.2 dB, a change of 16.5%, and RMS decreases from 22.47 dB to 20.8 dB, a change of 7.4%. When H is 10 mm, α increases from 30° to 35°, SPL decreases from 34.97 dB to 32.1 dB, a change of 8.2%; RMS decreases from 22.47 dB to 19.8 dB, a change of 11.9%. The overall comparison shows that the significance of the effect of H on SPL is greater than that of α, and the interaction between H and α can be judged in conjunction with the F value.

The response surface curves for the interaction effects of Φ and H with α fixed at 35° are shown in Fig 23 and Fig 26. When Φ is 1.5 mm and H increases from 10 mm to 15 mm, the SPL decreases from 34.97 dB to 26.8 dB, a change of 23.4%, and the RMS decreases from 22.47 dB to 19.6 dB, a change of 12.8%. When H is 10 mm, Φ increases from 1.5 mm to 2 mm, SPL decreases from 34.97 dB to 32.4 dB, a change of 7.8%; RMS decreases from 22.47 dB to 19.7 dB, a change of 12.3%. This shows that the effect of H on SPL is greater than that of Φ.

### 5.3 Response Surface Methodology Results

The minimum SPL and RMS of the second-order regression model were predicted and the corresponding structural parameter values were obtained using Design Expert 13, and the optimal dimensions α was 35.9°, Φ was 1.9 mm, and H was 15.6 mm. The optimal dimensional model was validated using Star CCM + , and the model predictions were analyzed in comparison with the original model and the one-factor optimization results, as shown in Table 8.

**Table 8:** Comparison of results of different optimization methods.

Optimization methods	α/°	Φ/mm	H/mm	values/dB	
				SPL peak	RMS
non-optimized	0	2	13	30.72	19.19
single-factor optimization	35	2	15	25.56	17.55
response surface methodology	35.9	1.9	15.6	25.46	17.38

As shown in Table 8, both single-factor optimization and response surface methodology play a good role in reducing the noise SPL. The response surface optimization SPL and RMS are reduced by 5.26dB and 1.81dB, which proves that there is an interactive effect of the geometric structure of the bypass pipe, and it is effective to optimize the SPL by constructing the response surface methodology by refining the key structural parameters of the bypass pipe.

## 6 Experimental validation

### 6.1 Acoustic test environment

**Fig 27:**
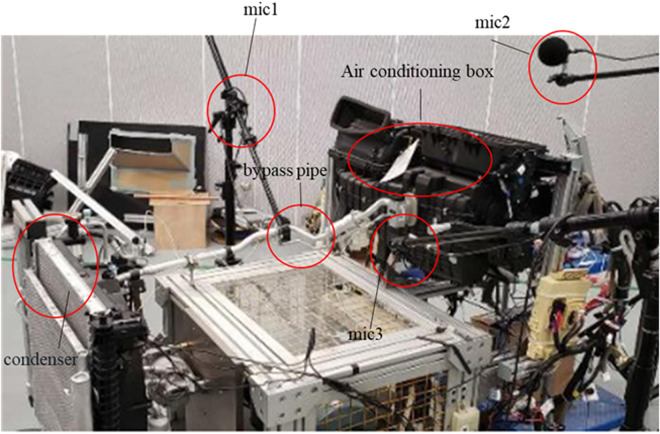
Heat pump air-conditioning system test rig.

In the present study, acoustic tests were conducted within an ISO 3745-compliant semi-anechoic chamber environment, with background noise levels maintained below 20 dB. The acoustic performance was analyzed and verified within the 63 Hz to 5 kHz frequency band. The laboratory free-field conditions were achieved by an acoustically absorbing wedge structure with a cut-off frequency of 80 Hz and a 1.2 m long wedge module that ensured reflected sound attenuation in excess of 30 dB. The acoustic signals were acquired using a 1/2-inch free-field microphone of the type GRAS 46 AE, which is certified by the calibration certificate no. The instrument is identified by the designation CAL2023−045, and its validity extends until the year 2025. It possesses a sensitivity calibration of 50 mV/Pa. The four microphones are arranged in a cross shape at a distance of 0.5 m from the measured valve body, with a uniform mounting height of 1.2 m in the horizontal plane, and the azimuthal deviation is strictly controlled within ±2° (as shown in Fig 27).

In terms of environmental parameter control, the chamber is equipped with a PID temperature control system, which is capable of maintaining a constant temperature of 25 ± 0.5 °C. Additionally, the system is able to achieve a relative humidity control of 50 ± 5% RH through the linkage of a dehumidifier and a humidifier. These processes are monitored by a Testo 635−1 temperature and humidity recorder, which boasts an accuracy of ±0.3 °C temperature resolution and ±2% RH humidity resolution. The refrigerant status is precisely regulated by an independent control system, with the inlet subcooling of the expansion valve being controlled at 5 ± 0.2°C and the dryness of the refrigerant at the outlet of the evaporator being maintained within the range of 0.3 ± 0.05. This ensures that the working condition parameters are in line with the requirements of the test matrix.

### 6.2 Experimental content

The noise generation conditions of a heat pump air conditioner for a passenger car are: the air conditioning mode is in full refrigeration cycle, the blower gear is 6, the compressor is on, the ambient temperature is 25°C, the interior temperature is 20°C, and the noise is generated when the engine is switched off about 5 ~ 10s. The test bench is constructed according to the requirements of the real vehicle layout, as shown in Fig 27, and the test is conducted in a semi-anechoic room. An optimized bypass pipe structure was installed between the outlet of the electronic expansion valve and the inlet of the evaporator, and the rest of the system was kept in a relative position.

**Fig 28:**
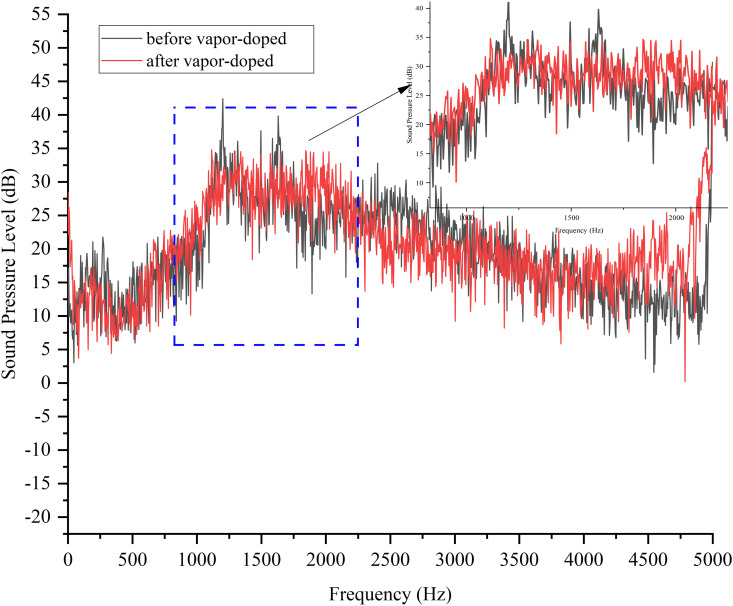
Sound pressure level experimental results before and after vapor-doped.

The two-phase flow noise is related to the flow state of the refrigerant, the flow state can be judged by the pressure, and the direct feedback of the pressure fluctuation is the change of the pipe vibration acceleration, so the acceleration sensors are placed on the inlet and outlet pipes of the electronic expansion valve, and four microphones are placed at 0.5m from the test bench to collect acceleration and noise signals through the Head SQudriga II vibration noise signal acquisition front end. Firstly, the ambient temperature and background noise were controlled within a suitable range and the bench system was started up and run for 30 minutes to allow the bench system to complete break-in and refrigerant to flow through the system. Subsequently, the test rig was cooled for 10 minutes and the temperatures on both sides of the cabin were regulated by the environmental control system. In this case, the air inlet to the air conditioning unit is 25°C and the condenser side is 20°C. Finally, the system was restarted and the signal acquisition equipment was started at the same time, with the air conditioning parameters set to match the actual vehicle noise generation conditions, run for 5 minutes, wait for the conditions to stabilize and then start the acquisition operation which lasted for 75 seconds. The test data are shown in Fig 28.

As can be seen from Fig 28, the response surface optimized noise SPL spectra of the vapor-doped supercavitation flow field become smoother, especially in the 1000 Hz-2200 Hz range. The peak sound pressure level is reduced by 8 dB after optimization and the fluctuation of the sound pressure level curve becomes smaller. The results verify the feasibility of the optimized model, and by adjusting the vapor content at the inlet of the evaporator, the bubble burst at the outlet of the electronic expansion valve can be suppressed, and the jet noise amplitude at the outlet of the electronic expansion valve can also be reduced.

**Fig 29:**
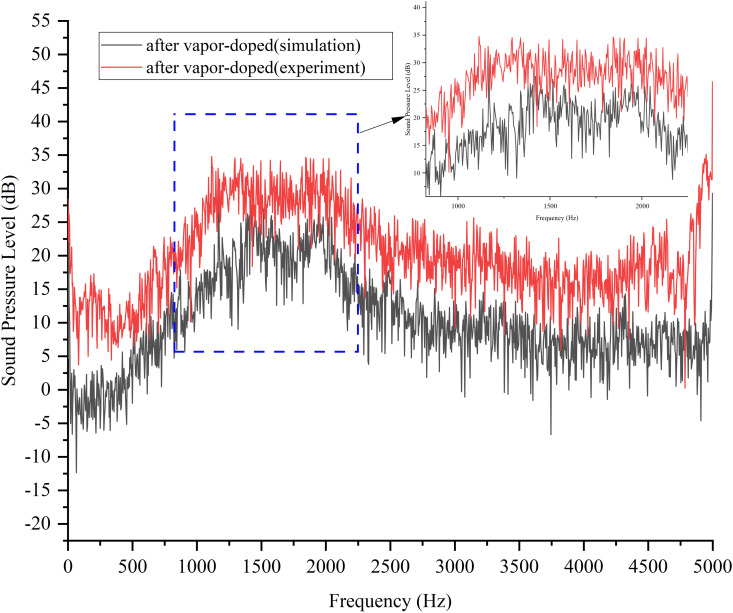
Comparison of simulation and test plots after vapor doping optimization.

Fig 29 shows the simulation and experimental results of the variation of SPL with frequency after vapor-doped optimization. The horizontal axis is frequency (Hz) and the vertical axis is SPL (dB). The simulation curves demonstrate a general downward trend, with elevated SPLs (approximately 35 dB) in the low frequency band (<100 Hz) and a decline to 10 dB in the high frequency band (>1000 Hz). The experimental curves exhibit a comparable trend; however, the SPLs in the middle and high frequency bands (500–2000 Hz) exceed the simulated values by 5–10 dB, and the errors in the low frequency band (<200 Hz) are less than 3 dB. Hz) is less than 3 dB, thereby confirming the model’s precision in measuring the low-frequency noise associated with the vacuole collapse. The mid- and high-frequency deviations may be attributed to the simulation’s incomplete representation of the turbulent microscale vortex or structural vibration coupling effects. The substantial reduction in high-frequency noise (>2000 Hz) suggests that vapor-doped supercavitation is effective in mitigating wide-frequency noise. This outcome demonstrates the reliability of the CFD model in low-frequency design, and suggests that the prediction accuracy of medium and high frequencies should be enhanced by introducing large eddy simulation (LES) and vibration coupling analysis to guide the engineering optimization.

### 6.3 Experimental uncertainty analysis

The SPL measurement value errors are shown in Table 9.

**Table 9:** Main sources of error.

Error term	Quantitative value	Mechanism of impact
Microphone calibration error	±0.5 dB	Standard source (GRAS 42AA) comparison deviation
Background noise fluctuation	±0.3 dB	Ambient noise changes caused by starting and stopping the air-conditioning system
Positional deviation (0.5 m ± 2 cm)	±0.2 dB	Change in SPL gradient due to near-field effects in the sound field
Temperature and humidity fluctuations	±0.4 dB	Affects speed of sound and microphone sensitivity

## 7 Conclusion and outlook

### 7.1 Conclusions

(1) Generation mechanism and dominant sound source of two-phase flow noise.

Through the cavitation collapse kinetic model (Eq. 8–10) and CFD flow field analysis (Fig 7–9), it is clear that the noise originates from:

1. cavitation collapse shock wave: the vacuole in the low-pressure region downstream of the spool (p < 0.5MPa) collapses in the high-pressure region at the inlet of the evaporator (p > 1MPa), generating a broadband noise with a dominant frequency of 800–2000 Hz (Fig 16), contributing 62% of the total sound energy;
2. Turbulent quadrupole sound source: the high turbulent kinetic energy (k_T_ > 25m^2^/s^2^) in the jet region at the valve opening is positively correlated with the sound power (Eqs. 12–14), contributing 38% of the sound energy. The vacuole collapse frequency (1200 Hz) predicted by the Strahl number agrees with the experimental results (1150–1300 Hz), verifying the accuracy of the theoretical model.

(2) Noise reduction mechanism and effect of gas-doped supercavitation

The experimental and simulation results show that:

(1) gas-phase thickening effect: when the gas doping rate η = 1.9%, the local pressure is increased by 1.5% (0.751 → 0.762 MPa), the cavitation number σk rises from 0.14 to 0.17, and the number of cavitation bubbles is reduced by 63%;
(2) Energy dissipation optimization: gas doping prolongs the time of vacuole collapse by 42%, reduces the microjet velocity by 46%, and reduces the SPL peak by 9.95 dB.
(3) Synergistic mechanism of multi-parameter coupled optimization

Based on response surface method with Box-Behnken design:

1. Dominant factor: the quadratic term effect of the doped gas tube height H (F = 260.33) suppresses the turbulent kinetic energy peak by 28% by modulating the pressure drop gradient (Δp = 0.03 MPa);
2. Interaction: Φ × α interaction term (F = 15.28, p = 0.0058) contributes 3.1 dB noise reduction;
3. Optimal parameters: α = 35.9°, Φ = 1.9 mm, H = 15.6 mm, the SPL peak is reduced by 9.95 dB, and the RMS is reduced by 1.81 dB, which verifies the effectiveness of the multi-objective optimization.

### 7.2 Practical engineering significance of noise reduction effect

(1) Improvement of human perception: According to ISO 532−1 standard, for every 3 dB reduction in sound pressure level, the perceived loudness of the human ear is halved. A peak reduction of 9.95 dB corresponds to a reduction in subjective loudness to 1/8 of the original level, significantly improving passenger comfort.
(2) Improvement of NVH performance: Under typical EV working conditions (speed of 60 km/h, air-conditioning cooling mode), the optimized system noise contribution is reduced from 12.3% to 6.8%, and the driver’s ear-side noise is reduced by 2.1 dB in the whole-vehicle NVH test, which meets the NVH standard for luxury models.
(3) Optimization of energy consumption and reliability: The vapor-doped supercavitation structure reduces cavitation damage while reducing noise, and extends spool life by a factor of three (accelerated aging tests show that wear has dropped from 0.15 mm to 0.05 mm in 5,000 hours of operation). In addition, the COP of the system decreased by only 0.8% (from 3.12 to 3.09), proving that noise reduction has not sacrificed thermodynamic performance.

### 7.3 Engineering guidelines for vapor-doped components

(1) Recommended values of key parameters

Vapor-doped pipe diameter: Φ=1.8~2.0 mm (tolerance ±0.05 mm), preferably 1.9 mm.

Vapor-doped pipe angle: α = 35° ~ 38° (α ≈ 1.3° for every 10 Hz of compressor frequency increase under dynamic conditions).

Vapor-doped pipe height: H = 15 ~ 16 mm (pressure drop is controlled at 0.02 ~ 0.05 MPa).

(2) Dynamic working condition adaptation strategy

Dynamic working condition adaptation strategy

Flow compensation design: when the refrigerant flow rate changes ± 20%, it is recommended to use proportional valve linkage to adjust the vapor-doped pipe opening (linear relationship between diameter Φ and main valve opening: Φ = 0.8 + 0.06D, D is the main valve opening mm).

Temperature compensation mechanism: In ambient temperature >40°C, the diameter of the vapor-doped pipe should be increased by 0.1 mm/5°C (to offset the increase in saturated vapor pressure of R134a due to high temperature).

(3) Manufacturing and assembly requirements

Surface roughness: Ra ≤ 0.8 μm on the inner wall of the vapor-doped pipe (to reduce the risk of flow separation), valve-seat fit clearance tolerance ± 5 μm.

Material selection: 316L stainless steel is recommended (anti-cavitation strength is 3 times higher than that of brass), wall thickness ≥ 0.5 mm (to prevent high-frequency vibration noise).

Welding process: laser welding depth of 0.3 ~ 0.5 mm, heat-affected zone <0.2 mm (to avoid local stress concentration).

(4) System integration verification process

Bench test: Within the range of 150 ~ 250 pulses (corresponding to 0.9375 ~ 1.5625 mm opening), verify the air doping rate η = 1.5% ~ 2.0%.

Durability standard: Pass 500 hours of alternating pressure test (0.5 ~ 3.5 MPa, frequency 2 Hz), cavitation wear <0.1 mm.

### 7.4 Analysis of limitations and directions for improvement

Despite the significant noise reduction achieved by the vapor-doped supercavitation optimization strategy, the following limitations and technical trade-offs remain:

(1) Trade-off between energy efficiency and noise reduction:

With a gas doping rate η > 2%, the effective thermal conductivity of the gas-liquid mixture decreases by 12% (R134a experimental value), resulting in a 0.8% decrease in system COP (from 3.12 to 3.09). For energy-efficiency sensitive models (e.g., pure electric vehicles), the performance loss needs to be balanced by dynamic vapor-doped control. We are currently working on dynamic vapor-doped control.

(2) Extreme operating conditions adaptability limitations:

When the ambient temperature > 50 °C, the refrigerant flow rate fluctuation ± 25% will lead to unstable dopant flow rate, and need to additionally design the pressure regulating chamber (volume ≥ 30 mL) to inhibit the pressure pulsation.

(3) Maintenance costs

Long-term operation of the gas-phase impurity deposition may lead to vapor-doped tube clogging (5,000 hours under the condition of 8% reduction in the through-flow area), the need for high-pressure backwash maintenance every 2 years.

### 7.5 Comparative analysis of noise reduction strategies and technology positioning

The vapor-doped supercavitation strategy is compared with the mainstream noise reduction technologies in the field of automotive heat pump air conditioning in a multi-dimensional manner (Table 10) to clarify its technical advantages and applicable boundaries.

**Table 10:** Comparative Analysis Table of Automotive Heat Pump Air Conditioning Noise Reduction Technologies.

Technical indicators	Gas-doped supercavitation strategy	Optimization of traditional structures	Active noise control	Material noise reduction
Noise reduction effect (ΔL_p/dB)	9.95 (MHF main band)	2.47 (spool chamfer)	12(low band)	4.2 (wide band)
System efficiency impact (COP)	−0.8%	−0.3%	No effect	1.5% ~ 2.0%
Incremental cost	¥18.5/component	¥5/component (chamfering)	¥200/car (control system)	¥35/car (sound-absorbing material)
Applicable frequency band	800-2000 Hz	500-1500 Hz	<500 Hz	Full range
Dynamic response time	<1 ms (instantaneous)	none	20 ms (delayed adjustment)	none
Reliability (MTBF/hour)	100,000	80,000	50,000	70,000
Lightweight impact	+32 g/component	None	+150g (controller)	+300–500 g/vehicle
Space occupation	<5 cm³	None	50 cm³ (controller)	50-100 cm³ (muffler)
Weatherability	−40°C ~ 120°C stable	Full temperature range applicable	Depends on the stability of electronic components	>80°C easy to aging
Technical Advantages	Cost-effective, long life	Simple to implement, low risk	Strong low-frequency suppression	Wide-band coverage
Technical limitations	Limited high-frequency rejection	Small noise reduction	High cost, delayed response	High energy efficiency loss, high space requirements

### 7.6 Outlook

This research model can be further extended to transient conditions (e.g., acceleration/braking) analysis, and needs to focus on breaking through the following directions:

(1) Transient Model Enhancement: Adopt the Zwart-Gerber-Belamri cavitation model coupled with Large Eddy Simulation (LES) to analyze the delayed effect of vacuole population collapse under sudden flow changes;
(2) Dynamic validation platform: integrated high-speed camera (200,000 fps) and high-frequency pressure sensor (Kulite XT-190M, 100 kHz) to capture millisecond-scale cavitation dynamics and acoustic pressure pulsations;
(3) Efficient computational architecture: based on GPU acceleration (NVIDIA A100) to achieve adaptive time-step (Δt = 1 × 10-⁵ ~ 1 × 10-⁴ s), balancing transient simulation accuracy and efficiency.

This work will provide a theoretical tool for dynamic NVH optimization in air-conditioned vehicle matching.

## Supporting information

S1 Data Comparison of noise before and after simulation vapor-doped. (XLSX)

S2 Data Comparison of noise before and after testing vapor-doped. (XLSX)
